# Analectic

**Published:** 1832

**Authors:** 


### MISCELLANEOUS INTELLIGENCE

### ANALECTIC, ANALYTICAL, AND ORIGINAL.

### ANALECTIC.

1. *An account of the Origin, Progress and Present State of the Medical School of Paris.*

We publish some extracts from an article in the American Journal of the Medical and Physical Sciences, on the subject of the system of Medical Education in France—a system by far the most perfect in the world. We regret that our limits do not permit the insertion of the whole article. Enough however will be presented to give a general idea of the system, and to inform our readers how immeasurably distant the best regulated institutions in this country are from the perfection of the Parisian school. F.

From this digression we turn to the main object of the present article, commencing with the course of study, which the aspirant to the doctorate must go through. It is necessary to premise, that the following remarks apply equally well to all the medical faculties of France. In order to be entitled to a diploma, the candidate must furnish certificates, proving that he has complied, prior to his commencing the study of medicine, with certain prerequisites; for example, that he has obtained the degrees of bachelor of letters and of bachelor of sciences. To receive the former he must have been examined, and have been found competent on, the following branches—Greek literature, Latin eloquence, Latin poetry, French eloquence, French poetry, philosophy, history of philosophy, ancient and modern history, ancient and modern geography.

To obtain the second of these degrees—that of bachelor of sciences, the candidate must have answered in a satisfactory manner on mathematics, natural philosophy, chemistry, zoology, botany and mineralogy. After obtaining those degrees, and presenting his certificate of birth, the written authorization of his father, or if he be a minor, of his tutor or guardian, to his engaging in the pursuits of the medical profession, a certificate of correct and moral behaviour; after presenting these, and furnishing a security, if his father or guardian do not reside in the place, any individual may present himself with a view of commencing the study of medicine, and is admitted to take his first inscription. These inscriptions consist in placing one's name on a register kept for that purpose, and receiving a certificate attesting the fact. This ceremony is gone through every three months, and in order to be entitled to present himself as a candidate for the doctorate, the student must have received sixteen inscriptions; from which it follows, that the term required by law for the prosecution of professional studies, before the degree of doctor in medicine, or doctor in surgery is conferred, is four complete years; unless, however, these studies have been prosecuted in a secondary school. In such cases, the term required is six years, and it is in the same way obligatory upon the students, previously to have obtained the degrees already noticed—of bachelor of letters, and bachelor of sciences.

Inscriptions received in one medical faculty are of equal value in all the other faculties of the kingdom, provided, however, they are accompanied with a certificate of correct behaviour, from the dean of the faculty, or from the academical council, where they have been received. The first inscription can only be taken out at the commencement of the scholastic year. The royal council authorizes it sometimes to be taken out in the quarter, commencing in January when satisfactory reasons for not doing otherwise have been alleged, by the student; but under no pretext is he allowed to commence his studies in the third quarter. The inscriptions must be taken out in regular succession; unless the reasons assigned for not doing so, are held satisfactory by the faculty. A student who takes out an inscription for one of his comrades, loses all his own. The inscriptions received as aspirant to the title of officer of health, either in a primary or secondary school, are counted for the doctorate, provided the candidate presents, before the 13th, his diplomas of bachelor of sciences and bachelor of letters. But the diploma of officer of health is not admitted as a substitute for inscriptions in a faculty, if the studies required for obtaining said diploma have not been made in a primary or secondary school. The courses of lectures given by medical societies cannot supply the place, in order to obtain inscriptions, of those given by legally established schools. Military surgeons of the second and third classes, who have been employed in the armies, can avail themselves of their services for the purpose of dispensing with the inscriptions; or if they have attended the medical lectures delivered in the military and navy hospitals of instruction, (but in no other hospital,) each of these years of study are received as equivalent for one spent in attendance on a special school. If a young man, after taking out a certain number of inscriptions in a faculty, is called upon for his services in the army, he cannot, on his return, avail himself of the inscriptions allowed him gratuitously for those services, however numerous they may be, for any other purpose than to complete those he was in need of, prior to his departure. Students residing in cities where faculties of medicine are established, cannot avail themselves of the studies they have made in hospitals, with private teachers, or even in the school, without having taken out the inscriptions corresponding to the period of those studies, unless they can prove, by means of certificates obtained from competent authorities, that it was impossible for them to comply with that regulation. Even under these circumstances, only four inscriptions are allowed them. Nevertheless, an exception is made in favor of those pupils who, though placed in this predicament, have gained premiums in the faculties.

In our last communication we stated, that the number of branches taught in the medical faculty of Paris amounted to sixteen. Unlike what occurs in the medical schools on this side of the Atlantic, and we believe, in the English and Scotish Universities; but in conformity with the plan pursued in Italy and Germany, the student is not at liberty, in France, to choose himself, the lectures he is to attend in each year. A regular distribution of the courses, the number of these necessary for each student to attend, and the order in which they are to be attended during each session, is fixed by the faculty, and publicly announced at the commencement of the scholastic year. To ensure the observance of this arrangement, each pupil receives a card, by which he is enabled to gain admittance to the lectures he is required to attend. Yet, though this be the case, no student is actually forced to attend the lectures, and all that he is required really to do, is to take out his inscriptions at the regular periods, and to go through the examinations in a creditable manner. If he does that, he may abstain altogether from the lecture room, and seek his knowledge where he deems best. Some years ago an attempt was made to compel the class of each professor to attend him punctually, by calling over the roll not less than twice a month. But this plan was soon abandoned; for the students, unwilling to submit to it, and determined to prevent its being put into operation, answered in full chorus to each name that was called, coughed, sneezed, and scraped their feet, so as to cover the voice of the professor.

The duration of the scholastic year is fixed at a meeting of the faculty, held prior to the opening of the course. The faculty decides on the same occasion, on the days and hours at which the different professors will deliver their lectures. But whatever be the duration of the full session, the courses are divided into two sets—the winter and summer courses. The former commences usually in the early part of November; the latter in the beginning of April, and continues until the end of August. The following will afford an idea of the distribution of the courses during the winter and summer sessions of the four years of the studies, drawn up by an arrete of the royal council in 1820.

First Year.—*Winter*.—Anatomy, Physiology, Chemistry.—*Summer*.—Medical Natural Philosophy, or Hygiene, Surgical Pathology, Botany.

Second Year.—*Winter*.—Anatomy, Physiology, Operative Surgery.—*Summer*.—Medical Natural Philosophy, or Hygiene, Pharmacy, Surgical Pathology, Surgical Clinic.

THIRD YEAR.—*Winter.*—Materia Medica, Medical Clinic.—*Summer.*—Operative Surgery, Surgical Clinic, Medical Pathology.

FOURTH YEAR.—*Winter.*—Medical Clinic, History of Medicine.—*Summer.*—Surgical Pathology, Legal Medicine, Clinic at the Hospice de Perfectionnement, Midwifery.

Since the period at which this arrangement was made, some changes, occasioned by the organization of the school. have been effected in the distribution of the courses. The following list, showing the courses delivered in the winter and in the summer sessions, though not quite satisfactory, since it does not indicate the lectures that must be attended in each year by the student, is copied from Almanach General de Medecine, for 1830.

*Winter Lectures.*—1st, Anatomy; 2d, Physiology; 3d, Medical Chemistry; 4th, Surgical Pathology; 5th, Medical Pathology; 6th, Operative Surgery and Dressings; 7th, Surgical Clinic; 8th, Medical Clinic; 9th, Obstetric Clinic.

*Summer Lectures.*—1st, Medical Natural Philosophy; 2d, Hygiene; 3d, Medical Natural History; 4th, Midwifery; 5th, Surgical Pathology; 6th, Medical Pathology; 7th, Pharmacologia; 8th, Therapeutics; 9th, Medical Jurisprudence; 10th, Surgical Clinic; 11th, Medical Clinic; 12th, Obstetric Clinic.

From this it will be perceived that all the clinics, as well as medical and surgical pathology, are taught both in winter and summer. These double courses are not, however, obligatory in both seasons, but have been arranged in that way with a view to accommodate those students who feel disposed to attend them during summer instead of winter, and who have thereby more time to devote to anatomy and the other branches.

We now pass to the subject of the examinations. Before presenting himself to these, the pupil must of course have complied with every prerequisite for graduation, and must in particular present the diplomas of his degrees in letters and sciences. Nevertheless, officers of health, who have served in the capacity of military surgeons, as well as those who have taken out inscriptions in a secondary or in a military medical school prior to the first of January, 1823, are dispensed with presenting the diploma of bachelor of sciences. The degree of master of arts obtained in a foreign university, cannot supply the place of that of bachelor of letters. Pupils who on the 1st of January, 1826, had more than one inscription, were allowed, until the 1st of January, 1827, the choice between two modes of examination. Agreeably to the old mode, the pupil who had taken out sixteen inscriptions was, at the expiration of the last quarter, allowed to offer himself as a candidate for the degeee of doctor in medicine, on submitting to five examinations and defending a thesis. Anatomy and physiology constituted the subjects of the first examination; pathology and nosology of the second; materia medica, chemistry, and pharmacy of the third. At the fourth examination the candidate was tried on hygiene and medical jurisprudence; while the fifth examination was appropriated to the medical or surgical clinics, according as the candidate aspired to the degree of doctor of medicine or doctor of surgery.

Agreeably to the new mode, the subjects of the examinations are arranged in the following order:

1st. *Examination three months after the eighth inscription.*—Medical Natural History, Medical Natural Philosophy, Medical Chemistry, and Pharmacologia.

2d, *Examination three months after the tenth inscription.*—Anatomy and Physiology.

3d. *Examination three months after the twelfth inscription.*—Medical and Surgical Pathology.

4th. *Examination three months after the fourteenth inscription.*—Hygiene, Legal Medicine, Materia Medica and Therapeutics.

5th. *Examination three months after the sixteenth inscription.*—Medical Clinic, Surgical Clinic, Midwifery.

Candidates who have undergone the examinations of the eighth, tenth, twelfth, fourteenth quarters, (trimestres,) are alone allowed to take out the tenth, twelfth, fourteenth, and sixteenth inscriptions.

From the 1st of November, 1829, every candidate without exception have been compelled to undergo their examinations agreeably to the preceding mode. These examinations are conducted by two professors and an aggrege. Each of them has an adjunct who supplies his place in case of his being absent. The examinations take place for four candidates at one time, their names being registered according to alphabetical order. They commence at one o'clock, and terminate at three; so that each candidate is examined during half an hour, or during ten minutes by each examiner. Whether the candidate be examined agreeably to the old or to the new mode, he is required to hand in, at the fifth examination, the details of six observations collected at the bed-side of the sick. Of these, four at least must have been collected in the clinics of the faculty, and their authenticity must be certified by the professor. Candidates for the degree of doctor in medicine present the histories of four cases of internal, and two of surgical diseases, while those who aspire to the degree of doctor in surgery, are required to furnish four of surgical, and two of internal diseases.

The candidates are classed, for their examinations, agreeably to the date of their inscriptions, and under no pretext is the name of the examiners communicated to them beforehand.

For the examination on anatomy and physiology, the candidate makes on the dead body an anatomical preparation previously designated to him. On a subsequent day he answers to several anatomical and physiological questions, having a bearing on the nature of the preparation he has made, and gives a demonstration on the skeleton of the different parts of osteology. At the examination on materia medica, chemistry, and pharmacy, the candidate answers demonstratively to the questions put to him on medicinal substances. The examination on internal and external pathology, (medical and surgical,) is conducted in Latin. It takes place at one sitting. The same holds in respect to the examination on hygiene and legal medicine, in which the candidate is required to write the formula of a report on a subject proposed at the moment.

At the examination on the clinics, questions proposed beforehand are drawn out by lots, (tirees au sort.) They have reference to fixed and well known practical cases. To these questions the candidate must answer in Latin and in writing. With this view he repairs to the school, at least three hours prior to the examination, and there prepares his answer without the assistance of any one. In presence of the examiners he answers *viva voce*, in Latin, to all questions which his own written answer may elicit. When the candidate aspires to the degree of doctor of medicine, a greater number of medical than surgical questions are proposed to him at this examintion. The reverse is the case when he wishes to obtain the degree of doctor of surgery. Under these circumstances he is moreover obliged to perform on the dead body all the operations required, in soft and hard parts, for the cure of the diseases which form the object of the examination.

It occasionally happens that a candidate is found incompetent on particular branches, and is required to attend to these forsome time longer. In such case this extension of studies, and the examinations consequent upon it, must always, unless by special leave, take place in the same faculty.

After expressing, by inscribing his name on a register kept for the purpose, his intention of defending his thesis, the candidate deposits the manuscript at the office of the administration, and the dean designates a president who examines and signs it, and becomes responsible for the principles and opinions it contains, in relation to religion, public order, and morals. If he considers the thesis suitable for forming the subject of the sixth examination, the president makes a report to that effect. Should this not be the case, his decision is referred to the faculty. He superintends the printing, signs the proof sheets of the essay, which cannot be distributed unless he certifies that all the formalities have been complied with. Should a thesis, when distributed to the public be found different from what it was in the manuscript submitted to the examination of the president, or should it have been printed before the manuscript had received the sanction and signature of the latter, it would, together with the sixth examination if the latter had taken place, be annulled. The candidate would be refused his diploma, and obliged, before obtaining it, to defend a thesis on another subject; after a lapse of time fixed by the royal council; and that too,independently of other academic punishments that may be incurred in consequence of any objectionable principles contained in the printed thesis.

The thesis is defended before the president of the board, several of the professors, and two aggreges. The act continues an hour. The president has no vote in the decision of the jury of examination, but may nevertheless take part in the deliberation. In case of an equal division of votes relative to the propriety of admitting the candidate, the opinion ofthose in his favor is adopted. Every certificate ofcapacity delivered by a faculty, must, before being converted into a diploma by the royal council, be approved by the rector, (whose office is filled at

Paris by the dean,) and the certificate of approbation refers as much to the moral conduct as to the capacity of the candidate. The rector has consequently the right of refusing a diploma to one who does not merit it. The diploma is given by the grand master, who may suspend the delivery, of it, referring this act to the council of state. He may also compel the candidate to go through his examination a second time.

Doctors in medicine who wish to obtain the title of doctor in surgery, and vice versa, are required to sustain a fifth examination, and defend a thesis on a surgical or medical subject, according to the nature of the degree they wish to obtain. They are at the same time obliged to pay one hundred francs for the fifth examination, one hundred and twenty francs for the sixth, or thesis, and one hundred francs for the seal of the university, making a total of three hundred and twenty francs. But a doctor in medicine is not allowed to present himself to the fifth examination for the doctorate in surgery, before having defended his thesis in medicine, and the examiners must be apprized of his intention, of obtaining the one or other of these degrees, in their letter of convocation.

When, in 1794, the schools of Paris, Montpellier, and Strasburgh, which, like all the other establishments of the kind, had been abolished a few years before, were reorganized, a professor and an adjunct were attached to each chair. In the progress of time, however, these adjuncts became assimmilated to the titulary professors. From this, it followed, that the number of these was doubled; and that, as the number of chairs was not increased, except at Paris, where a professorship of medical bibliography was created, there were, in fact, two professors for each chair. By the same law, (4th Dec. 1794,) the professors were directed to be appointed by the committee of public instruction, on the presentation of the commission of public instruction. By the general law on that subject, dated 11 Floreal, an x, (10th May, 1802,) it was directed that the professors should be nominated by government, from among three candidates; the first of whom was presented by one of the classes of the Institute, the second by the general inspectors of the studies, and the third by the professors of the school. This arrangement continued in force until the period of the organization of the university by Napoleon, on the 17th of March, 1808. By the decree of that date, it was ordered that, at the first formation of that gigantic establishment, the grand master should nominate to all the chairs in each of the faculties; but that at subsequent periods all vacancies occurring in them, should be filled by individuals selected after a public concours.

By the ordonnances of November, 1822, and February, 1823, which effected the dissolution of the old faculty, and the reorganization of the school of Paris; and by subsequent ordonnances, which occasioned similar changes in the schools of Montpellier and Strasburgh, a new arrangement was made in the board of teachers attached to these establishments, and in the mode of nominating them. This arrangement consisted in appointing two sets of teachers—professors properly so called, and aggreges, or adjuncts.

The mode of election by *concours* was rejected for the former; but admitted for the latter. This occasioned at the time, and for a long period after, much discontent among the members of the profession, and the medical journals were clamorous in their demands for the readoption of the concours for the selection of the professors themselves.

Such was the organization of the faculty and the mode of electing professors when the revolution of July, 1830, broke out. In our last communication we stated that by the ordonnance of Louis Philip, dated the 5th of October, which revoked the obnoxious ordonnances of 1822 and 1823, it was ordered that vacancies occasioned by the death of some of the old professors, who were reinstated in their chairs, should be filled, as formerly, by a public *concours*. It remains now to state, that the same formality was enjoined in all subsequent elections. This measure, which was demanded by a considerable majority of the profession, not only as a matter of right, because it had been established by law, and abolished illegally by an ordonnance, but also as the most proper to ensure the admission into the faculty of men of talents, and the exclusion of ignorant pretenders, was, however, opposed by some who preferred the mode by presentation—by others again who advocated the method of simple nomination by the academic council, by general election among all the physicians of the place, &c. But the partisans of these methods were few in number in comparison with those who advocated that by *concours*, a circumstance which doubtless contributed in confirming the government in the idea of prescribing the latter in all vacancies that might subsequently occur in the faculties.

But before we proceed any farther we must be allowed to correct an error which crept into our last article. It is there stated, that in consequence of the changes effected in the faculty of Paris by the ordonnance of October, 1830, the body of the aggrégés was necessarily suppressed. We should merely have stated that the *privileges* conceded to these supplementary professors—of being allowed to deliver private lectures and the only class eligible to professorships, both of which were violently opposed as unjustifiable monopolies and as preventing emulation, were suppressed by that ordonnance.

The body itself, thus shorn of its obnoxious privileges, and reduced in its functions, to supplying the place of absent professors, and to a participation in the examinations, was retained.

The following will present a full view of the regulations of the concours:

Section I.—*Composition of the Jury of the Concours.*

*Article 1st.* The jury of the concours is composed, 1st, of eight professors of the faculty of medicine of Paris; 2d, of four doctors in medicine or surgery, or academicians who are not professors of the faculty, and who are selected in a manner presently to be mentioned, in the Royal Academy of Medicine, in the Academy of Sciences, or among the physicians and surgeons of the hospitals.

*Article 2d.* The judges selected from among the professors, are,

1st, for the chairs of natural philosophy, chemistry, medical natural history, pharmacy, and materia medica; the professors attached to these chairs, and in addition to them the professors of anatomy, physiology, hygiene, and legal medicine.

2d. For the chairs of surgical clinic and surgical pathology, operations, obstetrics, of obstetric clinic, and of anatomy; the professors attached to these chairs, less one of surgical clinic, who will be excluded by lot.

3d. For the chairs of medical clinic and medical pathology; the professors attached to those chairs, and in addition, the professors of physiology, materia medica, and hygiene.

4th. For the chairs of physiology, hygiene, and legal medicine; the professors occupying those chairs, and the professors of anatomy, natural philosophy, chemistry, obstetrics, one of the six professors of surgical clinic and pathology drawn by lot, and one of the six professors of clinical medicine and medical pathology, also chosen in the same way.

If by rejection, (récusation,) or any other cause, one or several professors of the four preceding series are prevented from performing duty, substitutes will be provided from among the professors of the three other series.

*Article 3d.* The judges selected out of the faculty are as follows:—

For the chairs of anatomy, physiology, medical and surgical clinics and pathtology, obstetrical clinic, surgical operations, obstetrics, hygiene, materia medica, legal medicine, and pharmacy, four doctors in medicine or surgery, chosen by the academy of medicine in the corresponding section or sections of that body. Two of them must be selected from among the physicians and surgeons of the hospitals.

2d. For the chairs of natural philosophy, chemistry, medical natural history; four members of the Academy of Sciences chosen by that body, to wit, for the chairs of natural philosophy and chemistry in the two sections of natural philosophy and chemistry; for the chair of natural history in the three sections of natural history.

*Article 4th.* To these twelve titular judges will be added three supplementary ones, (suppléans,) two of whom will be selected from among the members of the faculty, and drawn by lot, and one chosen by the Royal Academy of Medicine.

These supplementary judges will attend at all the sittings of the concours, and will supply the place, the two first of the professors of the faculty, and the third the place of the judges not attached to that body, whenever these may be obliged to absent themselves during the continuance of the concours. Under no other circumstances are they allowed to take part in the deliberations of the jury.

*Article 5th.* The titulary and supplementary judges elect by ballot the president and secretary of the jury.

Section II.—*Of the Conditions required of Candidates.*

*Article 6th.* In order to be entitled to present himself as a candidate to a chair in the faculty of medicine of Paris, every individual must be, 1st, a Frenchman by birth or by letters of naturalization; 2d, full twenty-five years of age at the moment of the inscription; 3d, and either a doctor in medicine or a doctor in surgery.

SECTION III.—*Of the Trials of the Concours.*

*Article 7th.* The concours is composed of four kinds of exercises or trials:—

1st. The appreciation of the anterior claims of each candidate, made at an assembly of the judges, and at which the merit of his writings and services is fully discussed.

2d. A printed dissertation handed in to the jury twenty days before the opening of the concours, and the subject of which consists in general views on the disputed chair, and on the plan and method that should be adopted in teaching the particular branch.

3d. A written answer to a question drawn by lot, and which is the same for all the candidates, made in a close room, (a huis clos,) and during a space of time which is the same for all. The candidates afterwards come in rotation, and read their compositions at an assembly of the jury.

4. A lecture delivered after a day's preparation, on some subject connected with the object of the chair—each candidate drawing by lot the subject on which he is to lecture.

5th. A lecture delivered after three hours preparation, on a subject drawn by lot, and which is the same for all the candidates who can go through that trial on the same day.

*Article 8th.* The candidates for the chairs of clinic are exempted from these last mentioned trials, to which is substituted two clinical lectures delivered in the lecture room of one of the clinical hospitals of the faculty, after the visit of some patients selected by the jury.

*Article 9th.* The lectures are an hour in duration—they must be oral, and the candidates cannot make use of any other than simple notes.

SECTION IV.—*Of the Judgment of the Concours.*

*Article 10th.* Immediately after the last sitting of the concours, the judges assemble and elect by secret ballot, and by a majority of the whole, the successful candidate. Nine constitute a quorum. In case of equal division, the president has the casting vote. The judgment is immediately announced to the public.

*Article 11th.* The mode of ballotting is the same as for the election of members of the academy of sciences.

*Article 12th.* The candidate elected at the concours receives the investiture of the office from the grand master of the university.

Each of the professors received, and we believe continues to receive, a fixed salary of three thousand francs, besides ten francs for every examination at which he is present. The president at the last examination, (thesis,) receives fifteen francs. Every professor who is designated to be present at any *act* of the faculty, and absents himself without leave of the dean, is fined. If he absents himself without leave, and does not perform his duties at the school, he loses his salary during the whole time of his absence. Professors who in their lectures, discourses, or in their social intercourse, disregard the respect due to religion, morality, or government, or in any scandalous way compromises his reputation or the honour of the faculty, is referred by the dean to the academic council, and the latter, according to the extent of the offence, pronounces either his suspension from duty or his final expulsion. The punishments to which the professors are amenable, are, 1st, arrests; 2d, reprimand in presence of the academic council; 3d, censure in presence of the council of the university; 4th, transfer to an inferior office; 5th, suspension from their functions for a fixed period, with or without a total or partial privation of their emoluments; 6th, leave of retirement before the time of *emeritus*, with emoluments lower than the pension allowed under the latter circumstance; 7th, erasure from the list of members of the university. This is a most severe punishment, as it prevents the individual subjected to it from ever filling an office in any of the public departments.

Two weeks before the commencement of the scholastic year, each professor submits for the examination of the faculty the programme of his course. Each of the courses must be completed before the termination of the year, the faculty determining, before the commencement of the lectures, the duration of each course. The general programme is then published and posted up.

2. *Effects of Opium Eating.*—We took occasion not long since to remark on the singular analogy between the respective effects of opium and alcohol on the system, and to observe that it was the difference of the circumstances under which the two articles were commonly taken which produced so considerable a diversity in the symptoms resulting from them; and that as opium, when taken by an individual acted upon by external stimuli, as those of light and sound, often fails to produce somnolency, so alcohol, if swallowed under opposite circumstances, as in silence and darkness, proves directly soporific. Our opinion on this point has been recently confirmed by the result of some inquiries we have made of a person addicted to the habit of using laudanum in considerable quantity. The individual is a female forty years of age, who for some years has been in the habit of taking from half an ounce to an ounce of laudanum daily, for the sake of removing uneasy sensations apparently connected with a dyspeptic state of stomach.—She never experiences from its use the effect of sleepiness, nor are her hours of sleep at all affected by the very variable period at which she uses the drug. Her usual course is to take a dose a short time before each meal, and other doses at uncertain hours. Unless fortified in this manner, the stomach is so irritable as to be incapable of retaining food. The bowels are moderately, not excessively constipated, and as her habit is entirely sedentary, it is doubtful how far this circumstance is connected with the use of the drug. She is otherwise temperate in her habits.

To what extent, and in what manner, the use of opium is injurious to the animal economy, is a point which is yet undetermined, and which is the less likely to be settled, as the unhappy victims of this habit labor even more sedulously than those addicted to the use of ardent spirit, to conceal the fact from notice; while the effects being less marked, the concealment is much easier in the former case than in the latter. In fact, it not unfrequently happens that a physician attends a patient in the daily use of this indulgence, and remains entirely ignorant of its existence; while, on the other hand, the evil is of such limited extent compared with that of drunkenness, as to make it less desirable to bring it into public notice. The few statements which have been furnished us on this subject are either vague and general, as the accounts of travellers who have witnessed its effects in the East, or fanciful and exaggerated, as is the case with the Confessions of an Opium-eater. This last production, however interesting and highly wrought considered as a literary work, has too much of the marvellous and is too poetical to be consulted for sober facts. We see that attention has lately been attracted to this subject by a case which lately took place in England, the decision of which involved in some sort that of the question above alluded to. This arose out of a claim made by the representatives of a deceased nobleman in Scotland for the amount of insurance effected upon his life. The claim was resisted on the ground that the individual alluded to was an habitual opium eater, and that this fact was not made known to the company at the time when the insurance was effected. The particulars of the case are mentioned in the thirty-fifth number of the Edinburgh Medical and Surgical Journal, and we refer our readers to that authority for its details. It is sufficient here to mention, that during the two years that intervened between the signing of the policy and the death of the Earl, his daily portion was from two to three ounces of laudanum, and that at one time the allowance per diem was not less than forty-nine grains of solid opium, and an ounce of laudanum. The effect of this is somewhat variably stated by the evidence adduced on opposite sides; but the amount seems to be, that while under the influence of the drug he attended to business, saw and conversed with his friends, appeared cheerful, and exhibited no symptoms distinctly resembling intoxication: on the other hand, that his appetite became exceedingly impaired, that when he rose in the morning he was so stiff as to be unable to support himself, and that he was subject to depression and low spirits. Several physicians were examined and some of high eminence, on the general question as to the influence of this habit on the general health; but as it happened, not one of them was able from his own experience to pronounce it prejudicial. The jury found the insurance company liable. In consequence of this decision, and apparently with a view to make up the deficiencies of his own evidence, Dr. Christison has come out in the Journal above mentioned with his views on this subject. The doctor is unwilling to admit that the habitual use of this drug can be indulged in with entire impunity; but the facts which he adduces go to show that the injurious effects are often exceedingly slow in taking place; that when the constitution has become once inured to the poison, so that the aid of habit is fairly enlisted in defending the system, the nerves, as well as the stomach, will hold out for a wonderful period against its destructive influence. For the benefit of those who have not the opportunity of recurring to the article itself, we quote the following list of cases, which, even regarded as exceptions to a general rule, are sufficiently remarkable.

"1. A young lady of five-and-twenty has taken it largely for fifteen years. It was first administered secretly by her nurse to keep her quiet and save trouble; and the unhappy lady was subsequently compelled to keep up the practice for her comfort. She enjoys good health. 2. A female, a patient of mine in the Infirmary, a martyr to the rheumatism, took it for ten years previous to her fortieth year in the quantity of a drachm daily of solid opium. She then gave it up. Six months afterwards she was attacked with jaundice; subsequently she was several times severely ill of rheumatism; and she died in her forty-third year of consumption. This woman, however, led a licentious life from an early period. 3. A well-known literary gentleman, who has taken laudanum with some intermissions for twenty years, and occasionally to the extent of nine or ten ounces daily, has now attained his forty-fifth year. He is spare in form, looks older than he is, but is capable of undergoing a good deal of bodily fatigue, and enjoys tolerably good health so long as he takes sufficient exercise. His allowance when I had last an opportunity of conversing with him was about nine drachms of laudanum daily. 4. A lady in this city, after drinking laudanum to excess for upwards of twenty years, died about the age of fifty. No information could be supplied of the disease of which she died. 5. A lady of the same age takes about three ounces daily, and has used it for many years. She appears to enjoy good health. 6. A lady, about sixty years of age, had taken it above twenty years, and is in good health. 7. A charwoman, who had been in the daily practice of drinking two ounces of laudanum for many years, died at the age of sixty. The gentleman who has stated this fact, does not remember what disease she died of, although he dissected the dead body. 8. An eminent literary gentleman, I am informed, has been in the habit of taking laudanum since he was fifteen; and his daily allowance has sometimes been a quart bottle (twenty-six ounces,) consisting of three parts of laudanum and one of alcohol. Enormous as this dose may appear, I am assured this fact is well known to his acquaintances. He is about sixty years of age, and enjoys good health. 9. A lady of seventy, now alive, has taken about an ounce of laudanum daily for nearly forty years. She enjoys tolerable health, and every year travels great distances to visit her friends. 10. An old woman of eighty died a few years ago, at Leith, after taking about half an ounce of laudanum daily for nearly forty years; and she enjoyed tolerable health all the time."

The secret, no doubt, by which both alcohol and opium are so often rendered apparently innoxious, is to be found in their regular and systematic use, as distinguished from occasional indulgence in excessive doses. In regard to opium, this precaution is the more likely to be adopted, since an overdose is known to be attended with immediate danger; and those who acquire the habit are compelled to feel their way and increase the quantity only as the susceptibility becomes obviously diminished. The fitness for this kind of training, however, must vary in different individuals with other peculiarities of constitution; and there is little doubt but many pay with their lives the penalty of their imprudence, before the habit can properly be said to be established.

With regard to constipation, as an effect of this habit, it seems not to be more constant than the other ill effects which are attributed to its use; and it may be doubted whether in those who have become duly seasoned to its employment, this circumstance continues to constitute an inconvenience. The opium-eaters of the East are not compelled to use cathartic medicine, and in the case at least of those above mentioned, no laxatives at all appear to have been resorted to.

3. *Neuralgia successfully treated with the Cyanuret of Potassium.*—Dr. Lombard, of Geneva, has employed the cyanuret of potassium with success in the treatment of several cases of neuralgia. Dr. L. applies it by friction; he uses a watery solution in an ointment according to circumstances. The watery solution is of the strength of from one to four grains to the ounce of water, and the ointment is composed of from two to four grains of the salt to an ounce of lard.—The aqueous solution he considers in general, the most prompt in its effects.

Case I. *Facial Neuralgia instantaneously cured by the Hydrocyanate of Potash in Friction.*—A lady, of robust habit, forty-nine years of age, was a martyr to the most agonizing occasional attacks of pain in the space between the temporal region and the ciliary arch and maxilla. She used to scream violently in these torturing accessions, and sometimes lost all appearance of sensibility to such a degree that she had been supposed to be struck with apoplexy. Pulse 84; face rather flushed; no functional derangement. She was ordered to be rubbed with the aqueous solution, containing sixteen grains of cyanuret of potassium in four ounces of distilled water: it was rubbed on the forehead and cheek with a ball of cotton. The pain gave way almost instantaneously at the very first application, and seemed, as the patient said, to be rubbed away with the hand. A complete cure was effected by persevering a little while in the use of the remedy.

Case II. *Periodic Neuralgia removed by the Ointment of the Cyanuret.*—The cure in this instance was less prompt, but not less certain. A lady, thirty-eight years of age, experienced the severest pains in the temporal region and upper jaw of the left side: they came on regularly every morning at four o'clock; went on increasing in severity until about ten, and did not cease till four in the afternoon. In that interval she laboured under anorexia, fever, head-ache, &c. and was almost driven distracted. She was bled to twelve ounces to relieve congestion, and then the ointment was applied to the cheek and temple. Two grains to the half ounce of lard were at first employed, but the improvement was more rapid under the application of ten grains to two ounces. Lotions of the cyanuret were eventually used, and the cure was complete.

Case III. *Facial Neuralgia almost immediately cured.*—A lady, twenty years of age, suffered for several days, at the same hour, the most torturing pains in the orbital and supra-maxillary regions. Her face was much flushed, particularly on the affected side. Ten grains of the cyanuret were dissolved in four ounces of distilled water, and rubbed on with cotton. The application was quite successful.

Case IV. *Chronic Occasional Neuralgia similarly treated.*—A woman, of eighty, who had long suffered from irregular attacks of this complaint, was cured by lotions and frictions, compounded pretty strongly, and continued for some time.

The cyanuret of potassium is contra-indicated where the nervous affection is complicated with inflammatory action, discharge, &c. It is a useful remedy in non-inflammatory rheumatism.

In sciatic neuralgia it has not been successful—nay, it has been necessarily discontinued, on account of some unpleasant accidents which it occasioned.

In white swelling, attended with acute pain, poultices moistened with the solution had the effect of producing much comfort, though the continuance of their application had no promise of amendment in it.

It is on the whole inferred that the calming properties of this remedy are superior to those of any other known, and that it should always have a preference where inflammation does not exist. Lotions, with hydrocyanic acid, are by no means to be compared with it, for the acid is decomposed with facility, and scarcely to be used without danger.

The first application of the cyanuret in the above way is claimed by M. Buttigny and his brethren of Geneva, but it is disputed with them by Messrs. Robiquet, Villaume, and Bally of Paris—*Gazette des Hopitaux, Medical Gazette, Sept.* 1831.

4. *Excoriations of the Mammæ.*—Dr. Feist, of Bensheim, states, in the 4th volume of the *Gemeinsame Zeitschrift fur Geburtskunde,* that excoriations of the mammæ may be cured more promptly by a solution of corrosive sublimate than by any other means. He recommends two or three grains of the sublimate to be dissolved in an ounce of rose or distilled water. A little of this is to be warmed; a small piece of fine linen, several times folded, or a piece of lint wet with this, is to be applied so as to cover the excoriation. This application should be always made directly after suckling, and the breast must be carefully washed with tepid water or milk before applying the infant to the breast. Great care is of course always necessary in applying so powerful a remedy. When the excoriations are not very deep, Dr. F. says that they will be commonly cured by this means in a few days.

5. *Increased sensibility of the retina.* By R. Middlemore, Esq.—The term morbid sensibility of the retina applies to a variety of diseased conditions, which may or may not be attended with an organic change in the texture of the retina itself: for instance, the retina may possess an augmented sensibility to light, forming what is termed photophobia; it may be unusually sensitive to some particular colour, or to many colours; or it may be morbid as regards its sensibility only in reference to certain combinations of colour. Then, again, it is important to distinguish between increased and depraved sensibility, inasmuch as the former consists in an augmented sensation, from a natural degree of impress, whilst the other mistakes, distorts, and perverts the form, colour, magnitude, and distance of surrounding objects.—Each of these states of the retina is, in nearly every instance, symptomatic of disorder, disease, or irritation of some near or distant organ or texture, scarcely ever arising from any functional or organic defect in the retina itself as a primitive disease; but as I intend to confine my observations as much as possible to the increased sensibility of the retina to light merely, I shall only adduce the more ordinary causes of this single phenomenon.

The retina of strumous children has frequently a much increased susceptibility to light, without being combined with any change of texture: in the same way various morbid or irritable states of the uterine system lead to the production of this condition of intolerance of light; and the defects of this function to which I more particularly allude, are those in which the uterine secretion is altogether absent, or diminished in its quantity, to which must be added painful and irregular menstruation. I have seen very many cases of this description where the retina has been so intolerant of light that the eyelids have been closed during the whole ef the day with a spasmodic force equal to that which occurs in acute retinitis, so that any attempt at separating them has occasioned great pain, and induced severe spasm of the orbicularis palpebrarum, yet the eye itself has been perfectly free from inflammation, and quite natural in appearance, with the exception of a contracted state of the pupil. If such a condition of retina exists in combination with amenorrhœa, suitable remedies, directed to the torpid condition of the uterus, will, if they establish its natural function, quickly relieve the spasm of the orbicularis, and remove the augmented sensibility of the retina, and any attempt to restore the eye to its natural and healthy condition, under such circumstances, by local remedies, employed with a view of "deadening" the sensibility of the optic nerve, would be far from constituting the "most rational mode of treatment." A short time ago, I saw, in the absence of a medical friend, a young woman whose case is so fully illustrative of my views on this subject, that I shall briefly mention its outlines. She was a girl of a healthy appearance, and about nineteen years old, and had never yet menstruated, but suffered occasionally from pain in the loins and the lower part of the abdomen. The eyelids were forcibly closed; and from the frequent and powerful contraction of the orbicularis and corrugator muscles, they had acquired considerable power and thickness. The most careful attempt to examine the state of the eye occasioned extreme torment and profuse lachrymation, and was succeeded by a series of painful and spasmodic contractions of all the muscles of the eye-ball and lids; but there was no external inflammation, and no evidence of any deep-seated inflammatory mischief, although the pupil was extremely small and contracted. She had tried various local applications before I saw her without the slightest advantage, but was speedily cured by the use of Griffith's mixture and a few of the common pilulæ aloes c. myrrha. A young woman came to me a few days since from Westbromwich, with, to use her own words, a spasm of the eye-lids; and after examining the state of the eye, (one organ only being affected,) and observing its freedom from inflammation, I ascertained that she also was the subject of amenorrhœa; and although she has tried fomentations and collyria, of various kinds, for many months, I have little doubt of her speedy and perfect restoration by the employment of the constitutional treatment which the more important disease, (amenorrhœa) of which the condition of the retina is merely a symptom, requires.

Without entering further into the details of cases illustrative of the various causes of an increased sensibility of the retina, I may state, 1st, that it may be produced upon any source of gastric and intestinal irritation, the most frequent of which is, that condition of the mucous surfaces which occurs in strumous children, or is produced by the existence of worms; 2d, by general nervous excitement; 3d, by various morbid states of the sensorium; 4th, by various disordered, diseased, or irritable conditions of the uterine system; 5th, by inflammatory and other affections of certain parts of the eye; 6th, by disorder or disease of its own proper structure, and this is of all others the most infrequent cause of photophobia.

As regards the treatment of this increased sensibility of the retina, it will, of course depend altogether on the cause producing it; but as in nearly every instance it is but a symptomatic affection—a symptom of disease or disorder in some other part—local treatment, in the general, must be of a very subordinate character.

It is not, however, my intention to pursue this part of the subject in detail; for the adoption of such a course would be absolutely detaching one symptom of a disease from its associates for the purpose of distinct treatment; it would, in short, be making a separate disease of a symptom without any good or sufficient reason for so doing. The treatment of the scrofulous, the gastric, and the uterine photophobia, and, indeed, almost all its other forms, is, in fact, but the treatment of the disordered or diseased condition of that organ, or that state of the system, of which it is but an occasional symptom; and which must be conducted on the same principles which would guide us in the management of the same cases where no affection of the retina existed.—*Lond. Med. Gaz. July*, 1831.

6. *On Poisoning with Acetic Acid.*—In the *Journal de Chimie Medicale*, for August last, there is an account of some interesting experiments by M. Orfila, relative to poisoning by acetic acid. The following are M. Orfila's conclusions:—

1st. That the concentrated acetic acid is an active irritating poison, capable of speedily producing death in men and dogs when introduced into the stomach.

2d. That it occasions sanguineous exudation, afterwards softening and inflammation of the membranes of the intestinal canal, and sometimes even their perforation.

3d. That in most cases it produces a blackish colour, either general or partial of the mucous membrane of the stomach and bowels. This discoloration is the result of the chemical action of the acid on the blood, in fact, by its mixture with this concentrated acid, the blood, cooled and placed in a capsule, soon acquires the same tint.

4th. Common vinegar, in the dose of from four to five ounces, induces the same symptoms, and death in dogs of the common size, in from twelve to fifteen hours, unless it is vomited soon after being swallowed; in rather a larger dose it probably produces the same effects in man. If in opposition to this it is said that persons have swallowed a tumbler of vinegar without a fatal result, this doubtless depended upon the stomach of such persons being filled with food, and vomiting soon occurred.

7. *Opium.*—I have treated equal quantities of Turkey and English opium by the same process, and obtained twenty per cent. more morphia from the latter than the former; this would sanction the belief of the superiority of the English; which superiority, I think, is to be attributed to the careful manner in which it is prepared.

*I have invariably* found the flat pieces of opium to be the best, much more free from impurities, and have frequently found in the same case of opium the flat pieces to break with a short clear fracture, while the thick and round pieces were full of leaves and impurities, and I am thus always particular in selecting opium to reject the nodular pieces.

It is a matter of some moment to be in possession of the best preparation of a substance in such general use as opium. The preparation is, perhaps, the one which follows:

*Denarcotised acidulous Tincture of Opium.*—Digest 1 oz. of coarsely powdered opium in one pint of sulph. æther, s. g. 735 for ten days, occasionally submitting it to the influence of a moderate heat, until it ceases to act upon the opium, separate the opium and dry it, then digest in spt. vin. rect. 8 ozs. acetic acid fort. 2 ozs. aqua 6 ozs. for seven days, and filter. This preparation will be found to possess great advantages over laudanum and the black drop of the shops, to which it will be much preferable, inasmuch as it will be destitute of the stimulating principle, (narcotine,) which produces such distressing effects, and frequently forbids the administration of opium where it might otherwise be extremely useful. The addition of acetic acid will contribute much to increase the calming or sedative effects, which are most generally desired, and for which opium is particularly given. By its union with the morphia, it forms in solution the active sedative salt of opium, (acetate of morphia,) and differs only from the solution of the acetate of morphia of the shops, in its state of purity, and as the extraneous matter with which it is associated has no effect on the animal system, it may be considered as a good article, and should be preferred for general use, in consequence of being much less expensive. As this preparation will always possess uniform strength, and a like proportion of opium, it certainly deserves a conspicuous place among our pharmaceutical preparations, and justly merits to supercede entirely the common black drop of our shops, which is a very uncertain preparation, differing every where in activity from the indefinite and vague manner in which it is directed to be made, to say nothing of the worse than useless articles which enter into its composition, such as yeast, nutmeg, and saffron. The black drop owes its superiority over laudanum to the acetic acid of its composition, and to that *alone*, and it will be admitted by those conversant with the articles in question, that acetic acid exercises a most powerful inflence in modifying the effects of opium. This I can account for in no other way than by its uniting with the morphia, thereby rendering it much more soluble, and consequently facilitating its effects on the constitution, which are directly sedative, while the effects of opium in its natural state are stimulating.—*Carpenter's Essays on some of the most important Articles of the Materia Medica.*

8. *Acetate of Morphia.*—I would here remark, that the acetate of morphia, of the shops, is a sub-acetate, and is less active than the acetate or super-acetate, which, being a deliquescent salt, must necessarily be kept in solution; it is, therefore, requisite in making the solution from the sub-acetate, to add acetic acid rather in excess than under neutralization. The following is the formula I have adopted, which will make a handsome solution, and is a preparation that will keep:—

| | |
|---|---|
| Sub-acetate of morphia, - - - - - | 11 grs. |
| Alcohol, acidulated with twelve drops of acetic acid (pure *concentrated pyroligneous acid*), - - | 1 oz. |
| Distilled water, - - - - - - - | 1 oz. |

Dissolve the morphia in the acidulated alcohol, and add by degrees the water, and filter. Dose of the solution, from fifteen to twenty drops.—*Ib.*

9. *Piperine.*—Piperine has proved a valuable adjunct to quinine; equal proportions of each will act with much more energy than the whole quantity of quinine or piperine alone. Dr. Chapman informs us, he has met with much success in the treatment of intermittent fevers, by employing the following prescription:

| | |
|---|---|
| R. Quinine, - - - - - - | grs. X. |
| Piperine, - - - - - - | grs. X. |
| M. ft. Pill, - - - - - - | No. X. |

One to be taken every hour in absence of fever.

Oil of black pepper is much more active than piperine, one drop being fully equal to three grains of piperine. Three drops of oil of black pepper added to ten grains of quinine, will greatly increase the powers of this remedy. Oil of black pepper alone is a valuable stimulant in typhus fever, and is a valuable adjunct to many medicines.

From these experiments it is concluded, that the extract of pepper is not only one of the best succedaneums for the bark, but that it is even preferable to it, on several accounts.

1st. It never produces disturbance in the stomach or bowels.

2d. It never fails in producing a cure.

3d. Those who were cured did not in any one instance experience a relapse.

4th. It produces a regular alvine discharge, as well as the excretion of urine and sweat.

5th. None of those who were cured, experienced that sensation of langour, so common to a state of convalescence.—*Ib.*

10. *Salacine.*—This article has lately been introduced here, and as far as it has been yet used, has given the most entire satisfaction. Dr. Miller of Lancaster informs me that he has successfully treated several cases of intermittents, in which quinine appeared to have no effect, and which readily yielded under the use of salacine; and from the experiments which he has made with it, is fully of the opinion that it is a very valuable medicine, and more efficient than the quinine.—*Ib.*

11. *Oil of Cantharadin.*—A piece of paper which has been made to imbibe this oil, forms an excellent blister, which may be accommodated accurately to the shape of any part, however irregular. The vesication thus produced is so exactly circumscribed, that the blister formed corresponds with the sharpest angles which may be given to the paper employed. One drop is sufficient to make a blister the size of a quarter of a dollar, On such places where the skin is thicker or more solid than those which are less exposed and covered with clothing, it requires that the oil be applied two or three times in the course of one or two hours, or that the part to be blistered be covered rather more with the oil; this however will be seldom necessary, as blisters are most frequently applied on parts which do not require this particularity.

One ounce of this oil contains the vesicating properties of nearly one pound of cantharides. Its use is so mild that generally speaking it produces a blister without the least disagreeable sensation, except on those places where muscles, nerves, or tendons are in a state of compression. We trust an article possessing so many advantages will receive the sanction of the faculty.—*Ib.*

12. *Hermaphrodism.*—M. Rudolphi, in a memoir presented to the Academy of Sciences of Berlin, October 20th, 1825,, describes a case of Hermaphrodism of a very rare kind in the hnman species. It was m twith in the body of a child, who had died, as it was said, seven days after birth, but the developement would lead to the supposition of its being three months of age. The penis was divided inferiorly; the right side of the scrotum contained a testicle, the left side was small and empty. There was a uterus which communicated at its superior and left portion with a fallopian tube, behind which was an ovary destitute of its ligament. On the right side there was neither fallopian tube, nor ovary, nor ligaments, but a true testicle, from the epididymis of which there arose a vasa deverentia. Below the uterus, there was a hard, flattened, ovoid body, which when divided, exhibited a cavity with thick parieties. The uterus terminated above, in the parietes of this body, and at the right the vasa deferentia, without however penetrating into its cavity. Finally, at its inferior part there was a true vagina which terminated in a cul-de-sac. The urethra opened into the bladder, which was normal. The anus, rectum, and the other organs were naturally formed. M. Rudolphi considers the ovoid body, situated beneath the uterus, as the prostate and vesiculæ seminales in a rudimental state.—*Am. Journ. of Med. Sciences.*

13. *Extraordinary Abstinence.*—A curious instance of this is related in the *Memorial Encyclopedique et Progressive des Connaissances Humaines* for September last. The subject of the case was a farmer of Gaillac-Toubzac, arondissement of Muret, named William Granie, thirty years of age, who murdered his wife on the 5th of April last, in the most brutal manner, and was imprisoned at Nuret, where he murdered his bed-fellow. Granie was then transferred to the prison of Toulouse. On the 15th of April he determined to starve himself to death. On the 24th of April the first appearances of emaciation were perceived, and on the 29th he was effected with general tremors. On the 30th he had sufficient strength to break the padlock which fixed his hand-cuffs. On the 18th of May he was affected with violent oppression. The 6th of June deglutition became difficult; the 12th the pulse was 89; the 17th violent convulsions occurred, which terminated this slow agony, which had continued for sixty-three days. During this interval G. only drank at intervals a little water, and sometimes his urine. Although taciturn in prison, he constantly answered to questions in such a manner as to remove all idea of mental derangement.

In our sixth volume, p. 543, will be found an account of a case in which a man lived on water for 54 days.—*Ib.*

14. *On Inflammation of the Medullary Tissue of the long Bones.*—M. Reynaud, of Paris, the author of a paper which has just appeared with the preceding title, professes to have taken up the subject not so much with the view of giving a full treatise on it, as for the purpose of calling the attention of surgeons to a disease apparently of great frequency, and probably of much importance as the sequela of operations on the extremities. The reader may conceive the consequence attached by M. Reynaud to a thorough inquiry into all its relations, when it is added that in one of the great hospitals in the course of two years, every case of amputation of the thigh proved fatal, and in every instance was inflammation of the Medullary tissue of the remaining part of the thigh-bone found after death either singly or in conjunction with other morbid appearances.

The cases he has brought forward in illustration of the general principles which will be presently stated, are five in number; for, with a considerate attention to his reader's leisure and patience, not often met with among French pathological writers of the present day, he informs us that the numerous additional cases he might have likewise detailed, would not enable him to go further in his inferences, or to explain better the phenomena of the disease, than the examples which he has published, and of which we shall communicate the following short analysis. *Case I.* The first he considers an illustrative example of the inflamatory state of the medullary tissue in its lowest degree, such as is necessary to the production of callus on the end of the divided bone after amputation. The patient was a young man, whose arm was removed by M. Boux, on account of extensive disease of the elbow-joint. The operation was short, two ligatures only were applied, the stump next morning had healed by the first intention, and in a short time the ligatures came away, and cicatrization was completed. The Patient nevertheless continued languid, depressed and feverish; in a few days the left thigh and leg became painful and doughy; the right knee then became enlarged and obviously contained fluid; the fever in the mean time increased, and dry tongue, total prostration, and slight delirium ushered in death. On dissection there was found great serous infiltration of the left thigh, a little pus at the upper part of the tibia, the saphena vein thickened and red, and containing pus, the crural vein obliterated by a clot resembling in colour and consistence thick chocolate; purulent effusion into the right knee-joint, with redness of the synovial membrane; bloody injection of the dura-matral archanoid with a very thin layer of purulent-like effusion and serous infiltration of the pia mater; a fatty state of the liver. On examining the stump, the cicatrix was found firm, except at a small point only; a semi-fibrous layer rested on the end of the bone, firmest next the bone, and forming a plug over the medullary canal; around the edge of the bone a ring of bony matter was formed, and a smaller bony ring was also formed on the edge of the medullary canal; almost the whole medullary membrane of the bone was of a reddish-brown color; the periosteum was healthy; clots of blood were found in the sub-scapulary veins, where they end in the axillary vein; but the other veins, as well as the artery, presented a natural appearance.—*Case II.* The next is an instance of inflammation of the crural vein after amputation of the thigh, where the medullary tissue was found in a state of more advanced inflammation. The particulars of the case before death are not given; it is merely observed that amputation was performed on account of a chronic disease of the knee-joint, and that the patient died in thirty days. On dissection, there was found thin effusion of flaky pus on the archanoid; numerous purulent points dispersed throughout the substance of the brain; a few tubercles in the lungs; a purulent cavity in the spleen; the crural vein of the stump inflamed; the end of the bone denuded of its periosteum to a short distance upwards; the medullary tissue inflamed and purulent as high up as the separation of periosteum externally, and a considerable portion of the rest of it redder, denser, and less oily than natural.—*Case III.* This is an extremely interesting example of advanced inflammation of the whole medullary tissue of the bone. The patient, a lad, sixteen years of age, had a curved leg, and slight disease of the knee joint; and, together with his parents, insisted on having amputation performed to rid him of the incumbrance, although the surgeons of the hospital were averse to it. M. Boyer therefore amputated the limb at the usual situation above the knee-joint, and dressed the stump in the old French method. Every thing went on prosperously till the sixth day, on the morning of which M. Reynaud found him sitting in bed making caddis; but in the evening the countenance became yellow; delirium ensued during the night, and also diarrhoea; on the ninth day, the upper part of the thigh was much swelled, and on squeezing it, pus flowed profusely from the wound; acute pain in the stomach, hurried respiration, a short cough, fever, increased yellowness of the skin, and yellow tinging of all the secretions rapidly followed, and death took place on the tenth day. On dissection, the dura mater was found yellowish, and covered internally with a thin layer of lymp; a sero-purulent fluid was effused into the left pleural cavity; and the lungs were inflamed and infiltrated with pus. The veins proceeding from the stump were inflamed and contained pus as high up as the iliacs; the muscles near the flaps were infiltrated with a sero-gelatinous fluid; the periosteum was detached from the bone as high up as the greater trochanter; the bone was yellowish and surrounded by pus. The medullary tissue was inflamed and in a state of suppuration up to the very termination of the medullary canal.—*Case IV.* is an instance of extensive inflammation of the medullary tissue, without any other material diseased appearance in the body. Amputation of the thigh was performed by M. Roux on account of an old inveterate disease of the knee joint; the stump was dressed so as to heal by the first intention; and for fifteen days nothing occurred to disturb recovery. But then the stump became painful, the patient complained of the dressings being always too tight, and was very restless; subsequently strong fever set in, the breathing became frequent, and death supervened, without any particular symptom referable to the stump, except increasing and extreme tenderness, with diminution of the discharge. The appearances found on dissection were confined to the limb operated on. The soft parts were united except at one point, where there was an opening down to a purulent cavity round the end of the bone. The bone was denuded of its periosteum two inches up in front, and half an inch higher behind; above this it adhered to a thin layer of recently formed bone; and still higher it was still connected with the original bone, but more loosely than usual, and without any appearance of uniting vessels when it was stripped off. The extremity of the medullary tissue formed a brownish-white, spongy mass, infiltrated witb pus, and plugging up the cavity in the bone; above this plug there was a purulent cavity extending as high as the level of the trochanter—the pus lying in contact with the bone posteriorly, but separated from it anteriorly by a layer of tissue infiltrated with pus: and the cavity communicated outwardly by a small aperture. The cells of the spongy tissue of the bone above the medullary cavity were lined by a deep red membrane, and filled with pus. The pus was every where very fetid. The substance of the bone was unusually thick in front and thin before; its outer surface, instead of being smooth, shining, moist, and slightly rosy, was dry, white and dull; and its texture, as was seen when it was broken across, appeared unusually white and presented none of the red spots which are seen in a healthy bone after the laceration of its connecting blood-vessels.—*Case V.* is a similar instance of very extensive and advanced inflammation of the medullary tissue, united with various other morbid appearances. Amputation was performed on account of white swelling of the knee-joint. An attempt to heal the stump by the first intention failed; and profuse suppuration took place; but methodical compression gradually produced almost complete union. From the second day, however, violent fever set in, cough with thick expectoration followed, diarrhœa next appeared, and then yellowness of the skin before death. On dissection, the dura mater was found yellowish in color, and lined internally by a pseudo-membranous effusion; the left lung in the incipient stage of carnification, the right much gorged, and a purulent cavity at its base; and the pericardium containing some serum and a thin layer of lymph on both its surfaces. The flaps of the stump adhered partially. There was a purulent cavity at the end of the bone. The femoral artery contained pus towards its divided extremity. The femoral vein was throughout its whole course thickened, surrounded with brittle cellular tissue, lined with a pseudo-membrane internally, and filled with pus. The end of the bone was denuded of its periosteum to a short distance upwards, and the rest of this membrane was more easily stripped off than usual; but the surface and internal structure of the the bone, except just at its extremity where the periosteum had been separated, was of the natural smoothness and redness. The lower end of the medullary tissue was prominent, black, and purulent, and it was in a state of fetid suppuration throughout the whole cavity of the bone. In the general summary with which M. Reynaud concludes his paper, he observes that the frequency with which inflammation of the medullary tissue has been met with in the long bones after amputation, shows that there is some connexion between the disease and the injury done to this tissue by the saw, and he therefore throws out the hint whether it is not advisable to use some precautions for rendering the incision through the cavity of the bone smoother than it is commonly made, and likewise to cover it more closely with the soft parts of the flap. He considers himself justified in inferring from his cases, that the stripping of the periosteum from the bone is produced by an advanced state of inflammation of the medullary tissue, and that the extent of the one is generally conformable with that of the other. He is not satisfied he is yet able to point out the symptoms of the disease he describes, but says he would be inclined to suspect it wherever there is a general doughiness of the stump without external redness or other signs of inflammation—when a large quantity of matter may be squeezed out of the stump, which cannot be accounted for from the extent of the remaining sore—when there is acute, deep-seated pain, felt during the first dressings, and accompanied with extreme tenderness of the stump—and when there is yellowness of the stump, acidity of the breath, and general fever.

There appears little doubt that the subject of the author's paper has not hitherto been sufficiently attended to by surgeons, and is deserving of more notice. Some allusion to it has been made by M. Ribes, in the article Necrosis in the Dictionnaire des Sciences Medicales, and more lately by M. Blandin in the article Amputation in the Dictionnaire de Medecine et de Chirurgie Pratiques.—*Ed. Med. and Surg. Journ. Oct. and Arshives Generales, June*, 1831.

15. *Tetanus from Inflammation of the Spinal Cord*—An interesting case of this is related by M. Combette, in the *Archives Generales*, for June last. The subject of it was a healthy elderly woman, accustomed to field labor. She was seized without any apparent cause with acute pain in the loins, along the back, and almost immediately afterwards with a general and extreme rigidity, by which she was deprived of the use of her limbs. Five days afterwards she was admitted into the hospital St. Antoine, at which time there was a general contraction of the muscles, semiflexion of the forearms, with extreme difficulty in extending them, extension of the limbs with impossibility of bending them, and constant closure of the jaw, extreme difficulty and pain in speaking, difficult deglutition, redness of the features, suffusion of the eyes, fulness and hardness of the pulse, considerable frequency and some labor of respiration, and perfect preservation of consciousness. She was immediately and freely bled. Next morning her state was unaltered; and she screamed aloud when she was touched or any attempt was made to move her. The blood was buffy. Stimulant draughts were now given, with a drachm of laudanum, blood was withdrawn from the loins by cupping, cataplasms were applied to the back, and opiate friction used over the body generally. In the evening no change had taken place, except that deglutition had become much more difficult. At midnight the breathing was laborious and accompanied with mucous rattle and frothing at the mouth. Sopor now appeared interrupted, however, by screams when she was meddled with. The pulse continuing full and strong, and the countenance flushed. She was again freely bled, after which she expressed herself relieved. Her attendant then urged her to take one of her draughts, which she was very unwilling to do; she at length yielded; but immediately the contractions were much increased; violent convulsions ensued, and in this state she expired.

On dissection, thirty hours after death, there was found great rigidity of the extremities; vascularity of the cerebral membranes, and somewhat more redness of the cerebral texture than is natural; much serum in the canal of the spine; the spinal membranes, and particularly the inside of the dura mater, of a bright rose-red color; the surface of the cord covered with a net-work of vessels; softening of the anterior part of the cord, so as to present no resistance to the knife, and to form indeed throughout its whole length, a soft semi-liquid pulp of a lively rose color, especially in the cervical and lumbar regions, where some spots of extravasation were also seen; no deviation from the healthy state of the posterior part of the cord; no particular appearance at the origins of the anterior or posterior nerves; the pneumogastric nerves and cervical ganglions of the sympathetic nerve in a healthy state; the lungs gorged, especially at their base and posteriorly; the mucous membrane of the stomach of a pale yellow tint, with some red points here and there; the other internal viscera healthy.

16. *Small Pox.*—During the last eighteen months M. Louis has had the opportunity of examining the bodies of fifteen patients who died of small pox. In eleven of these he found in the bronchia either a false membrane, or a collection of pus or blood. In one-third of the cases there was a morbid development of the glands of Brunner forming exanthematous pustules. In no case were the elliptical plates altered. —*Gazette Medicale, June* 25*th,* 1831.

17. *Pustules in the Intestines caused by the internal administration of Tartar Emetic.*—M. Guerard says that he has met with, in two persons treated by large doses of tartar emetic, pustules similar to those produced on the skin by the application to it of that article. These pustules occupied a considerable portion of the intestinal canal; there existed besides no sign of dothinenteritis. The stomach was healthy.—*Ibid.*

18. *Aneurism of the Right Axillary Artery cured by Tying the Subclavian Artery.*—The subject of this case was a man sixty-three years of age. The tumor presented the section of an oblate spheroid, of about the size of a large orange; it extended from the clavicle into the axilla, and was attended with considerable unremitting pain almost utterly precluding sleep. The disease was attributed to a hurt received some time before; on careful examination, no disease of any other artery or of the heart was discoverable. The operation was performed on the 17th Dec. 1830.

"Sixty minims of laudanum were given immediately after the operation and which dose was repeated an hour or two afterwards, but without producing any considerable relief. The same night, the patient complaining of increase of pain in the wound, with considerable difficulty of deglutition, and some dispnœa, and a severe pain extending from the left hypochondrium to the right shoulder, he was bled to the extent of oz.xviij. and which operation was repeated the following morning, in both instances, with the greatest possible relief. He was of course restricted to a rigidly antiphlogistic diet and regimen, and the bowels relieved by repeated doses of a saline aperient, combined with antimony, preceded over night by an alterative aperient pill.

"From the above date, little remains to be observed, as to the mere history of the case, excepting that the above symptoms, though decreasing in violence, continuing from time to time to recur, were obviated, or reduced by various modifications of the same means; while, in addition, local painful affections were relieved successively, by the employment of warm spirituous fomentations, liniments, dry friction, the use of flannel, and the last ten days by the warm bath.

"The last ligature came away on the twenty-ninth of January and there now remains only the most minute moisture on the spot from which it escaped."

The operator and narrator of this case, W. Bland, Esq. attributes its successful issue in a particular manner, to the very early and repeated employment of the lancet, the use of spirituous fomentations to the limb, and the support given to it, by means of a broad flannel sling, by which the arm was steadily supported without sustaining any disagreeable pressure: this, in a case of unavoidable long duration, though apparently trivial in itself, was a matter of great moment. There is yet another circumstance, he adds, to which I shall beg to call attention: the careful, but gentlest possible expulsion, by means of pressure, of as much of the discharge as practicable, from the wound, each time that it is dressed; by which the danger to be dreaded from the unpropitious direction of the external opening of the wound, (which instead of being in a depending situation, is at all times almost perpendicular above its fundus), is in a great measure, if not entirely obviated.

There was a very slight expectoration of blood, on the 5th of this month, (February) but which was immediately obviated by bleeding, and has neither returned nor evinced any tendency to return.—*Lond. Med. and Surg. Journ. Oct.* 1831.

19. *Is the Meat of Diseased Animals unwholesome?*—The following remarks on this subject, from the *Gazette Medicale*, for 20th of August last, though far from satisfying us of the innoxiousness of the meat of diseased animals, are nevertheless worthy of consideration. The subject is an interesting one, and should receive more attention than has hitherto been bestowed on it.

Among the investigations which the central council of health for the department du Nord has included in its report for 1830, to the prefect of Lille, there is a question which appears to us of very great interest, namely, whether the meat of kine attacked with tuberculous affections proves injurious to the health of individuals eating it? The facts which led to this inquiry occurred recently in the Lille butchery; some of them are as follows:—In the middle of last January, a cow brought to market was suspected of being effected with the aforesaid disorder,, and the police having been informed of it, summoned Messrs. Pommeret, a veterinary surgeon, and Simon Pers, a merchant butcher, both considered skilful in their avocations. After having examined the animal with great care, M. Pommeret declared it to be affected with the tuberculous disorder, which affection was limited to the lungs and pleuro-costales, and appeared to have no connexion with other parts of the system: he was convinced that the animal in question, although slightly affected with tubercules, might be used as aliment, without producing any inconvenience to health.

Simon Pers, the butcher, declared that the cow had its lungs and sides covered with small pimples, (boutons,) which constituted the disease called leprosy, on which account he thought its meat should not be exposed to sale, but buried, as not being fit aliment for man.

After this discordance in the opinions of two persons, both regarded as expert, the police called upon a third, who gave it as his opinion, that as the tuberculous affection was confined to the lungs and pleura, all other parts of the system appearing sound, the sale of the meat should be permitted, which was accordingly done.

A few days previous, a similar case occurred. A cow having been pronounced to be affected with a leprosy and gangrene, was ordered to be buried. Messrs. Pommeret and Loiset, veterinary surgeons, repelled the charge of leprosy, none of the peculiar symptoms of which were found in the cow when examined, and which, besides, are only met with in swine. The designation of corrupted, (pourri,) which had been given to the animal, was considered by them as absurd and ridiculous, as they only recognized upon it a tuberculous affection, without any trace of other disease, and decided that the meat of the animal might be used as food without any inconvenience, and that permission to sell it should be given, which was accordingly granted.

These two facts go to prove, that at Lille, in the course of a few days, two cows, recognized as unquestionably laboring under the tuberculous disorder, have been used as nourishment by many hundreds of people, without causing any complaint. It is also well established that this disorder is of more frequent occurrence than is generally supposed, especially in winter. It is fair to infer from this, that the meat of animals may be eaten without risk, whatever disease they may have when killed? The author of the report replies to this question by adducing the following facts:—

In all the large towns, and more particularly in Paris, dogs, and especially the valuable wild animals in the royal menagerie, are fed almost solely upon the flesh of horses.

Now, many of these horses are not only sick when slain, but are brought to the place after having already died of diseases; all are cut up alike, and made use of without giving rise to any inconvenience.

During the revolution, the professors of the school of Alfort, near Paris, caused a great number of horses, affected with farcie and glanders, to be taken into the forest of Vincennes, and there slain. The flesh of these was all eaten as fast as they came, by the inhabitants of the neighboring villages, who, nevertheless, remained free from any disease.

In the year 1737, the following fact was communicated to the Institute, by M. Hamel. A herd of cattle on its way from Limousin, stopped at an inn at Pitiviers, in Gatinois; one of the finest not being able to go further, was sold to a butcher, who killed it at the inn. The butcher's boy having placed his knife between his teeth, his tongue swelled, and he died five days afterwards, with a general gangrene. The innkeeper, who received a cut on one of his fingers, was attacked with a swelling on his arm, and died at the end of a week. His wife, who had received some of the blood upon the hand, had a tumor formed on this part, and recovered with difficulty. Finally, a surgeon, who had opened one of the tumors, and placed his lancet between his wig and forehead, was attacked in this part with an erysipelas, which lasted a long time. Notwithstanding all this, said Hamel, all the meat of this animal was sold, and chiefly to the richest families; more than a hundred persons who eat of it, both roasted and boiled, found it excellent, and no one experienced the slightest inconvenience.

A dog, after having bitten seven cows, died a short time after with confirmed madness. Other dogs which he had bitten, were killed on showing indications of the same disease. The cows mentioned soon exhibited symptoms of madness, notwithstanding which, they were butchered and distributed to customers, without either the milk, which had been drawn up to the time they were killed, or the meat having occasioned the least complaint from any of the inhabitants of Montargis, the village where this circumstance occurred.

In 1814, the allied troops carried in their march large flocks of cattle which they had pillaged. These having been over driven and badly attended to, were all seized with intense inflammation of the stomach, intestines, and liver. This disorder became contagious, and spread its ravages through the whole country, traversed by the army, especially at Paris and its environs. But the meat of none of the animals which died or labored under this disorder, was suffered to be lost, all of it having been eaten by the strangers, citizens, soldiers, and lower classes: it was even consumed in the hospital. Neverthe less, there were no bad effects upon the health of individuals observed, and on the contrary, the typhus, which had preceded this epidemic, disappeared.

In 1815, the epidemic which occurred at the commencement of summer, continued till January, 1816. During six months of this epizootic, the allied troops received in their rations no other meat than that of animals that had labored under typhus. On no table was any other meat eaten, but such as came from sick animals, and yet no persons were incommoded.

We have extracted from the report of the members of the Central Council, a case of each of the diseases most common among herbivorous animals: these having been reported by persons worthy of credit, we conclude with the authors of the memoir, that the reprobation attached to animals killed in a state of disease, and the repugnance universally manifested to eating their flesh from a persuasion of its being noxious to health, are altogether prejudices, which, for the interest of trade and the general welfare of man, should be done away. This is likewise the opinion of Messrs. Huzard, Darcet, Chaberet, Flandrin, Dupuy, and of a host of other savans, who have given particular attention to matters relating to public hygiene.

20. *The Use of the Trephine in Epilepsy.*—The following interesting case has been furnished us by Professor Dudley, being the sixth of the sort which has occurred in his hands. The other five cases were related in his paper on "Injuries of the head," published in the first number of this Journal. The success of this practice establishes two important principles in Surgery;—1st, That the brain will bear severe mechanical irritation for a great length of time, without fatal disorganization; and 2dly, that the use of the trephine under such circumstances may restore the organ to its former healthy condition. The cases of Mr. Cline, first published, we believe, in the paper just referred to, bear only a slight resemblance to those of Prof. D., and are not meant by him, or by Sir Astley Cooper, who has since noticed them, to establish those principles so valuable in practice.

Mr. ——— received a gun-shot wound on the head in the month of March, 1832. On examination next day, his physician took from the wound a number of small bones, when, by reason of an injury done the dura mater, some brain escaped. So soon as the bones with the disorganized brain were removed, he was dressed, and at the expiration of two months he was thought to be well. The patient states, however, that a slight discharge continued to issue from the wound, and after some months Epileptic convulsions, with a great derangement of the general health, ensued. It was then discovered, on examination, that the matter issued from the surface of the brain, and that the cranium appeared to be diseased. Under these circumstances he came to Lexington for assistance, his friend having furnished the preceding narrative.

On his arrival here his general aspect was that of an individual, who had suffered greatly from derangement of the cerebral and chylopoietic functions. A cicatrix of two and a half inches in length, on the central and posterior portion of the right parietal bone, pointed out the original injury.

On two points of the cicatrix were discovered small sinuous orifices, from whence was discharged an unhealthy pus. By the aid of a common probe diseased bone was detected.

The trephine was applied in the direction, and on one side of the original fracture. So soon as the segment of bone was removed by the trephine, isolated portions of bone were discovered beneath the dura mater, in a cavity of some dimensions occasioned by the absorption of the brain. Three of these, amounting in size to the thumb and finger nails, were removed, together with a morbid growth from the surface of the wounded dura mater. Simple dressings were then applied, and renewed occasionally for the week, when the patient was discharged, free from all embarrassment, both in the corporeal and intellectual functions.—*Transylvania Journal of Medicine.*

21. *A Case of Hereditary Hæmorrhagic Tendency.* By Dr. Hughes, of Simpsonville, Ky.—In the IVth vol. of this Journal, page 518, I gave an account of a case of Hereditary Hæmorrhagic Tendency, ascertained to be universal among the male members of the family in which it exists; the females being, at the same time, as universally exempt from it. Since the publication of this case, I have met with one of the same character, in another and entirely different family, a succinct history of which I propose now to give, simply for the purpose of calling the attention of the profession more particularly to this singular disease, for such it should undoubtedly be considered.

On visiting the house of a respectable farmer of this neighborhood, my attention was directed to the case of a youth ten or twelve years old, which appeared to be rheumatic, and which was so pronounced. The correctness of my opinion was called in question by an old lady present, who was herself a member of the family, and intimately acquainted with the history of the case. On further inquiry I ascertained it to be one of hereditary origin, the rheumatism being only the sequel of another affection to which the boy had been subject from infancy, viz. hæmorrhage. Learning that the disease was common in every branch of the N. family, of which that of my friend Mr. P. was one, I inquired particularly concerning it, when the following facts were communicated:

1st. That spitting, vomiting and purging of blood; bloody urine; bleeding at the nose; extravasations of blood among the muscles and integuments of the body generally, especially of the extremities, producing dark discolorations and swelling, attended frequently, after a few days' continuance, with obtuse pain and stiffness, and copious and obstinate hæmorrhage from very inconsiderable incisions, on whatever part of the body they are made, have been exceedingly common among the male members of the connection.

2d. That the Hæmorrhage, whenever it has manifested itself, has been invariably attended with rheumatism to a greater or less extent.

3d. That the slightest sprains or contusions have generally been followed by rheumatism of the part.

4th. That the majority of the males, who have arrived at old age, have been much disabled by rheumatism.

5th. That on the approach of old age, the tendency to hæmorrhage has been less manifest.

6th. That a considerable number of the males have died in infancy and childhood.

7th. That deaths immediately from the loss of blood have been frequent; several resulting from the employment of the lancet, some from accidental wounds, others from various internal hæmorrhages, and two of the number, simply from the application of blisters—"*the blisters*" in the language of my informant "*drawing blood instead of water.*"

8th. That, of the two diseases, hæmorrhage and rheumatism, the former has always maintained the priority.

9th. That the females, though in no instance sufferers from this predisposition, have, nevertheless, invariably transmitted it to their offspring.

And 10th. That the predisposition in question can be satisfactorily traced as far back as the fourth and fifth generation.

We present the above as facts, upon the authority of several intelligent and highly reputable members of the family to which they relate; a personal acquaintance with whom, enables us to repose the utmost confidence in their communications.—*Ib.*

22. *Analysis of the Sulphur Springs at Nashville.*—The water of this Spring was first analyzed by the late Prof. Bowen. It has since been examined by other chemists, and is found to contain the following ingredients:

*Gases.*

1. *Carbonic acid.*
2. *Sulphurated hydrogen.*

*Solid Ingredients.*

1. *Muriate of Soda,* (abundant)
2. Muriate of Magnesia,
3. Muriate of Lime,
4. Carbonate of Lime,
5. Sulphate of Lime,
6. Sulphate of Magnesia.

In composition it corresponds very closely with the famous Harrogate springs of England, the most abundant substance in which is muriate of soda.

The use of this water has been beneficial in dyspepsia, liver complaints, and other chronic visceral diseases.—*Ib.*

23. *A Class of Abortives.*—There exists a very strange notion among most people, that there is, in our materia medica, a class of medicines with the power of producing abortion. No such class most assuredly has ever found place there, nor is any medicine known which possesses this power. Slight as the causes are which occasion miscarriage in persons strongly predisposed to it, there is no article which exerts sufficient influence over the uterine apparatus, to bring about this condition where the predisposition does not exist. No fact is probably better established than this, and why it is that certain articles have a reputation for such efficacy, it is difficult to imagine.

A controversy on this subject has attracted our notice in an English journal, and, in the course of it, we remarked particularly some very just views expressed of the precise efficacy of the secale cornutum, a medicine of great value, the medicinal virtues of which were originally discovered in this country, (by Dr. Prescott, we believe, of Newburyport,) and which is but just beginning to be well understood abroad.

Because ergot exerts so decided and immediate and unquestionable an influence over the efforts of the uterus during parturition, an impression has gone abroad, even among medical men, that it is capable of *inducing* uterine contraction de novo. The fact is that ergot has in all probability no effect over this organ, except during paturition. This sentiment was expressed in the second volume of this Journal, and from subsequent observation, we are induced to believe it will prove correct. It receives confirmation from the universal failure of medicine to induce pain, *after the completion of labor*, and from the extremely various and contradictory accounts of its supposed effects *before its commencement.* It is also confirmed by analogy, as set forth by Dr. Venables in the controversy alluded to.

"I know," says he, "that this drug is often administered to quicken the tardy and languid operations of the uterus, and no doubt, in some instances, with advantage. But there is a wide difference between assisting, and wholly preventing the natural functions of the organic system. The languid uterus may be excited to a more vigorous performance of its natural functions by a proper and judicious exhibition of such a medicine; but from what analogies can we infer that a natural operation is to be perverted or arrested, and an unnatural one substituted in its stead?"

Certain it is that some females, who have had reason to adopt every method they supposed to promise any thing toward producing this effect, have undergone severe and repeated vomiting, drastic purging, and various other operations, without success:—preferring even death to the inevitable consequences of a failure in their object, they have swallowed the most disgusting and powerful drugs—oils, essences, and decoctions—to the imminent hazard of life; and yet, after all been compelled to suffer disappointment, and submit, however unwilling, to the just punishment of their crime. Cases of this kind show us the difficulty, if not impossibility, of bringing on uterine contraction by any known medicine, where a predisposition to it does not exist. A like view of the subject has been taken by most authors.

"Every woman who attempts to promote abortion, does it at the hazard of her life."—*Bartley.*

"There is no drug which will produce miscarriage in women who are not predisposed to it, without acting violently on their system, and probably endangering their lives."—*Male.*

"It has frequently occurred that the unhappy mother has herself been the sacrifice, while the object intended has not been accomplished."—*Dr. G. Smith.*

"But we must conclude that there is no medicine, or abortive means, which always produce abortion, and nothing but abortion; there is none which does not endanger the lives of the mother and infant."—*Ryan*, p. 154.

"Now we shall have occasion hereafter, to show that medicines, internally administered, can seldom produce abortion."—*Paris and Fonblanque*, vol. iii. p. 90.

And again—

"From a very early period attempts have been made to devise means of producing abortion, by the administration of certain drugs, which were considered as capable of acting specifically upon the womb, and of occasioning the exclusion (expulsion?) of its contents. It would be idle to enumerate the various substances which have, at different times, been employed for such a purpose, not a few of which were derived from the fertile sources of credulity and superstition; and yet we are bound to admit, that upon this occasion, at least, credulity has proved a blessing to mankind, by suggesting the substitution of a harmless amulet, or an inefficacious drug, for an application of extreme violence and danger, and perhaps of death. *The physicians of the present age disclaim the existence of any specific class of abortives;* but we are ready to admit that the administration of violent medicines, by *involving* the *uterus* in the *general shock* thus given to the system, will occasion abortion, *provided there exist* at the same time *a certain predisposition* on the part of the female: should this latter condition, however, be wanting, the *poculum abortionis* may, by the violence of its operation, *destroy the life of the unhappy mother*, or very materially injure her, without *accomplishing* the object for which it was administered."

Burns, too, after speaking of the secondary effect sometimes attributed to strong cathartics, which induce tenesmus and inflammation of the stomach and bowels, takes care to add, that "it cannot be too generally known that when these medicines do produce abortion, the mother will seldom survive their effect."

The above authorities are all cited by Dr. V., and, in addition to them, he brings the evidence of his own experience, which is the evidence of an accoucheur well skilled and long practised in every branch of the obstetric art.—*Boston Med. and Surg. Jour.*

24. *Tenacity of Vegetable Life.*—Mr. Houlton produced a bulbous root to the Medico-Botanical Society, which was discovered in the hand of an Egyptian Mummy, in which it had probably remained for two thousand years. It germinated on exposure to the atmosphere; when placed in the earth it grew with great rapidity.—*Ann. de Chim.*

25. *On the Identity of Smallpox and Cowpox, and on a mode of inducing the Vaccine Pustule in the Cow at pleasure.*—In our last, we offered some account of the researches of Dr. Sunderland and others on the mode of producing the variolous or vaccine disease in the cow. The following are the conclusions to which Dr. S. has arrived, and which we offer in order to present a clear and explicit view of the result of his inquiries on a subject of deep interest to the profession and the public.—*Bost. Med. and Surg. Journ.*

"1. This discovery is new; for, although many have suspected the identity of smallpox in man and cowpox in the cow, and have in consequence performed inoculation with the matter of both, yet no one has previously ascertained the possibility of transmitting the contagion to the cow in the gaseous form, so as to decide the question beyond all doubt.

"2. The desire of Physicians and governments to discover cowpox in cows, in order to revive the vaccine lymph, is more than fulfilled by the discovery of a simple method of engendering cowpox in the cow at will.

"3. Jenner's discovery of the protective power of vaccination, hitherto imperfect, is now perfected, because the hitherto unknown nature and origin of cowpox are laid open.

"4. All previous uncertainty regarding the quality of vaccine matter, its degeneration, the loss of its protective property, and the like, must now cease, because we have obtained a clear insight into the nature of cowpox, and can lay down a substantial theory of its operation.

"5. This discovery must tend to widen the boundaries of physiology, pathology, and therapeutics, since it shows how the subtle contagion of smallpox, so hostile to the nervous system of man, may be conveyed in the aeriform state from him to the cow, excite in that animal a similar disease, but in doing so, be changed by the special constitution of this class of animals into a permanent contagion of a different kind.

"6. An instructive lesson may be drawn from this discovery how the poison of diseases in the gaseous form may be communicated to the lower animals, and according to the difference in their constitution engender diversified products, which may then be used as a protective means against the diseases from which they originated. Such, for example, may be subsequently proved of scarlet fever, measles, yellow fever and plague.

"7. It is now clear why in recent times smallpox has been seldom or never seen in the cow. For the cowpox of the cow arises merely from infection by the variolous exhalations from men recently affected with smallpox, and coming in contact with the cow. As epidemics of smallpox have been rare during the last thirty years, cows could seldom be exposed to infection, and have therefore seldom exhibited the disease."

26. *Influence of Atmospheric Electricity on the Eyes.*—We see peculiar appearances in weak and morbidly sensitive eyes, before the breaking out of a violent storm, showing the positive influence of an atmosphere which has now attained its maximum of electricity; and I am acquainted with several persons who are able, from a certain premonitory feeling of their weak eyes, to predict a thunder-storm infallibly, a considerable time beforehand. As every experienced oculist is convinced of the bad effects of an atmosphere of this kind, he will never extract the cataract during the approach of a thunder-storm.—BEER, *Lehre von den Augenkrankheiten*, 72.

